# Line-Field Confocal Optical Coherence Tomography (LC-OCT) for Skin Imaging in Dermatology

**DOI:** 10.3390/life13122268

**Published:** 2023-11-28

**Authors:** Flora Latriglia, Jonas Ogien, Clara Tavernier, Sébastien Fischman, Mariano Suppa, Jean-Luc Perrot, Arnaud Dubois

**Affiliations:** 1 DAMAE Medical, 75013 Paris, France; 2 Laboratoire Charles Fabry, Centre National de la Recherche Scientifique, Institut d’Optique Graduate School, Université Paris-Saclay, 91127 Palaiseau, France; 3 Department of Dermatology, Erasme Hospital, Université Libre de Bruxelles (ULB), 1070 Anderlecht, Belgium; 4 Department of Dermatology, Jules Bordet Institute, Université Libre de Bruxelles (ULB), 1070 Anderlecht, Belgium; 5 Groupe d’Imagerie Cutanée Non Invasive (GICNI) of the Société Française de Dermatologie (SFD), 75008 Paris, France; j.luc.perrot@chu-st-etienne.fr; 6 University Hospital of Saint-Etienne, 42100 Saint-Etienne, France

**Keywords:** optical coherence tomography (OCT), line-field confocal optical coherence tomography (LC-OCT), confocal microscopy, 3D reconstruction, dermoscopy, skin imaging, dermatology, artificial intelligence

## Abstract

Line-field confocal optical coherence tomography (LC-OCT) is a non-invasive optical imaging technique based on a combination of the principles of optical coherence tomography and reflectance confocal microscopy with line-field illumination, which can generate cell-resolved images of the skin in vivo. This article reports on the LC-OCT technique and its application in dermatology. The principle of the technique is described, and the latest technological innovations are presented. The technology has been miniaturized to fit within an ergonomic handheld probe, allowing for the easy access of any skin area on the body. The performance of the LC-OCT device in terms of resolution, field of view, and acquisition speed is reported. The use of LC-OCT in dermatology for the non-invasive detection, characterization, and therapeutic follow-up of various skin pathologies is discussed. Benign and malignant melanocytic lesions, non-melanocytic skin tumors, such as basal cell carcinoma, squamous cell carcinoma and actinic keratosis, and inflammatory and infectious skin conditions are considered. Dedicated deep learning algorithms have been developed for assisting in the analysis of LC-OCT images of skin lesions.

## 1. Introduction

Skin cancer is the most commonly occurring cancer in humans, with an incidence that has steadily increased worldwide over the last decades [1]. Despite recent advances in therapeutics, the early recognition and complete removal of the cancerous tissue before the onset of deep invasion or metastatic disease is crucial [2]. The standard diagnostic procedure in dermatology begins with a visual examination of the skin surface, often assisted by dermoscopy to observe relevant features of the lesions otherwise not visible to the naked eye [3,4]. If a lesion is considered suspicious, a biopsy is taken, and the tissue is processed for histological examination and grading using optical microscopy. The result of this time-consuming procedure is that the majority of all skin biopsies result in benign diagnoses [5]. Moreover, tumors can be missed when they are still at an early stage of development [4]. Given these major medico-economic issues, non-invasive imaging techniques allowing for “in vivo histology” have been developed [6]. Obtaining deep, high-resolution images of the skin non-invasively should enable real-time diagnosis, reducing the number of unnecessary biopsies and limiting the risk of missing malignant lesions when lesions are very numerous or poorly visible. Non-invasive imaging techniques would also improve surveillance and lesion mapping during surgery and follow-up examinations. The main techniques currently used clinically for in-depth skin imaging are ultrasonography, reflectance confocal microscopy, optical coherence tomography, nonlinear optical microscopy and, more recently, line-field confocal optical coherence tomography. Alongside these techniques, optoacoustic imaging is poised to become a powerful tool in dermatology, as witnessed by the rapidly growing number of biomedical and clinical studies reported [7].

Ultrasonography (US) has become a reliable adjunctive tool in dermatology [8]. US can be divided into conventional US (frequencies < 20 MHz) and high-frequency US (frequencies from 20 to 50 MHz). The former is widely used in dermatologic oncology for both the pre-operative staging and follow-up of melanoma patients. The optimal frequencies for the visualization of the upper layers of the skin are 15 MHz or higher. The higher the frequency, the better the resolution. However, employing higher frequencies results in decreased depth of US penetration. In typical clinical US systems (DUB SkinScanner75, Taberna Pro Medicum, Lüneburg, Germany, or SkinScanner, Dermus, Budapest, Hungary), the penetration can reach a few millimeters, but the resolution is limited to ~30 µm (depth) × 100 µm (lateral). An improvement in the resolution can be achieved, but the penetration is then severely degraded. Imaging at the cellular level deep into the skin is impossible with US.

Reflectance confocal microscopy (RCM) is an optical imaging technique, which allows for the analysis of the skin with a nearly histological resolution (~1 µm), i.e., at the cellular level (Vivascope, VivaScope GmbH, Munich, Germany). Real-time images obtained by RCM are oriented parallel to the skin surface (en face sections). RCM has been applied in the clinical field, in particular for the diagnosis of melanocytic lesions where it has been proven to increase the diagnostic accuracy when coupled with dermoscopy [9,10,11,12] and shown to significantly reduce the number of necessary biopsies [13]. Beyond its application in skin oncology, confocal microscopy can be useful to delineate indications for inflammatory and infectious skin conditions. Limitations of RCM are the relatively low penetration (200–300 µm) because of strong light scattering, the interpretation of the images oriented perpendicularly to the conventional histological sections, and the necessity of image mosaicking to increase the field of view.

Optical coherence tomography (OCT) is an interferometric imaging modality that was initially introduced in ophthalmology [14] and has become a standard technique in this field [15]. The first use of OCT in dermatology was demonstrated in 1997 [16]. OCT can create in vivo vertical sections and/or horizontal (en face) sections of skin with a resolution of a few micrometers. The penetration depth of OCT in skin tissues is ~1 mm, and the resolution is ~7 µm (Vivosight, Michelson Diagnostics, Maidstone, UK). The possibility to evaluate OCT images in a vertical sectional view makes it easier to compare them with histological sections as opposed to RCM. OCT has a rather large lateral field of view, ranging from 2 to 10 mm. The biggest potential of OCT in dermatology has so far been in the diagnosis, delineation, and treatment of non-melanoma skin cancers, especially basal cell carcinomas (BCC) [17,18,19,20,21]. Pigmented lesions, on the other hand, continue to pose great challenges in OCT imaging, the diagnosis of malignant melanoma using OCT having not being achieved mainly due to insufficient image resolution.

Non-linear optical microscopy (NLM) is a high-resolution imaging modality based on nonlinear interactions of light with tissues [22]. Compared with US and OCT, NLM offers a better spatial resolution, similar to that of RCM. Advances in nonlinear (multiphoton) excitation microscopes with contrast mechanisms such as second harmonic generation and stimulated Raman scattering further allow for the visualization of skin morphology and function based on molecular-level signatures of biological molecules. Major limitations of NLM are the orientation of the images (en face sections) and the small size of the field of view (350 µm × 350 µm with the DermaInspect system, Jenlab GmbH, Berlin, Germany). The relatively weak penetration (~200 µm) is also a limitation. The cost and size (not handheld) of the device (DermaInspect), compared to OCT and RCM, is also prohibitive.

Line-field confocal optical coherence tomography (LC-OCT) is an optical skin imaging technique introduced in 2018 [23]. Combining the principles of RCM and OCT, LC-OCT has lower penetration depth than conventional OCT but improved penetration depth compared to RCM. It offers improved image resolution compared to conventional OCT, approaching that of RCM. Moreover, LC-OCT can generate vertical and horizontal sections in real time and three-dimensional (3D) images in seconds. This article reports on the LC-OCT technique and its application in dermatology. The principle of the technique will be explained, and the latest technological innovations will be presented, including the incorporation of a dermoscopy modality. The technology has been miniaturized to fit into an ergonomic handheld probe for easy access to any skin area on the body. The performance of the technique in terms of resolution, field of view, and acquisition speed will be reported. LC-OCT images of various skin lesions, including skin cancers, will be presented and discussed. The potential of dedicated artificial intelligence (AI) algorithms for LC-OCT image analysis will be shown.

## 2. Materials and Methods

### 2.1. The LC-OCT Imaging Device

#### 2.1.1. deepLive™

The LC-OCT technique is based on a combination of OCT and RCM with the illumination of the skin with a focused line of light [23]. A vertical section image (a B-scan) is obtained from several depth-profiles (A-scans) acquired in parallel by a line-scan camera. Vertical sections can be obtained in real time by scanning in depth at a frequency of a few Hertz, enabling the focus of a microscope objective to be dynamically adjusted. Cross-sectional images in a horizontal plane (en face sections) at an adjustable depth can also be obtained by laterally scanning the illumination line focused on this plane [24]. Finally, 3-dimensional images can be obtained from a stack of horizontal (en face) sections [24]. In addition, images of the skin surface can be acquired simultaneously with the LC-OCT images using an auxiliary imaging system incorporated in the LC-OCT device.

**Figure 1:**
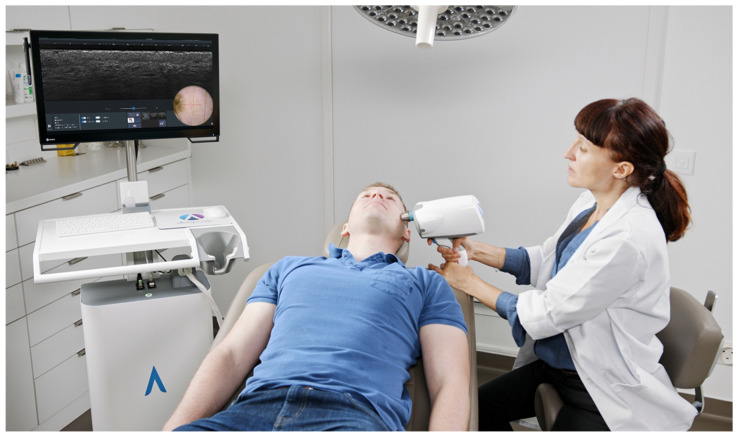
Line-field confocal optical coherence tomography (LC-OCT) device (deepLive™) commercialized by DAMAE Medical.

The LC-OCT technology has been industrially developed by DAMAE Medical. Since the company was founded in 2014, several generations of LC-OCT devices have been designed and developed following technical improvement plans. A first clinical demonstrator, composed of a microscope-shaped LC-OCT device, was developed in 2016 and released for investigative use only [23]. Following design and engineering studies, a miniaturized probe device was developed in 2018 incorporating LC-OCT technology. This device was CE-marked in class I for clinical research purposes, and 2 units were manufactured and installed in key-opinion leader (KOL) dermatology practices. Scientific research and technological experiments allowed for the extension of LC-OCT technology to 3D imaging [24]. A new device was then developed to integrate this latest innovation, leading to the release in 2019 of a pilot series of 10 device units installed in KOL practices. This new version was also CE-marked in class I for clinical research purposes. Finally, following a new iteration of technical improvements and design robustification, the first commercial version of the product, named deepLive™ (see Figure 1), was CE-marked in class I and released in 2020. The industrialization of the device supported the development of commercial activities of the company in the European dermatology market. In 2023, due to the evolution of the European Union medical device regulation and based on clinical evidence generated by clinical studies conducted with KOL partners, deepLive™ was CE-marked in class IIa with the intended use of assisting dermatologists with skin cancer diagnostics. To date (November 2023), DAMAE Medical has installed over 50 devices worldwide, mainly in Europe (France, Germany, and Italy), as well as in the USA.

A button and a scroll wheel on the deepLive™ probe handle allows the user to select the imaging mode (vertical, horizontal, or 3D) and to adjust the lateral/depth positions in the vertical/horizontal real-time imaging modes. Clicking on the button starts image recording. DAMAE Medical has developed user-friendly software connected to the imaging device. A patient file can be created by registering their demographic information and indicating the lesion location on a designed body mapper. The recorded LC-OCT images with their associated dermoscopy images can be reviewed. Image stacks can be displayed as texture-based volume renderings or orthoslices. For specific needs, various custom image processing algorithms are available to facilitate image review and analysis. Additionally, deepLive™ software V1.6.1 allows for the registration of clinical suspicions, LC-OCT diagnosis, patient management, and histological diagnosis to facilitate patient follow-up. The complete typical patient examination procedure with deepLive™ is described in detail in [25].

#### 2.1.2. Technical Description

**Figure 2:**
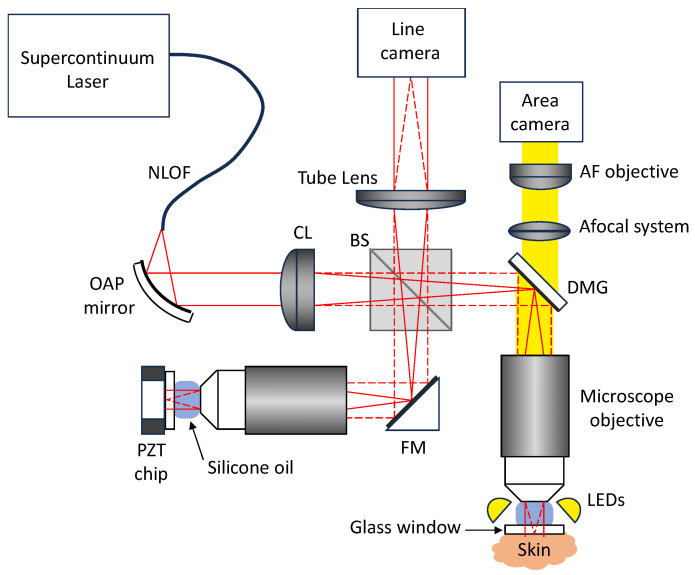
Schematic diagram of the LC-OCT device. NLOF: non-linear optical fiber; OAP: off-axis parabolic mirror; CL: cylindrical lens; BS: beamsplitter; FM: fold mirror; DMG: dichroic mirror galvanometer; LEDs: light-emitting diodes; AF: autofocus; PZT: piezoelectric.

The experimental setup of LC-OCT is shown schematically in Figure 2. It is based on a two-beam interferometer with a microscope objective in each of the two interferometer arms (sample arm and reference arm). A supercontinuum fiber laser (SuperK EVO, NKT photonics, Birkerød, Denmark) is used as a broadband spatially coherent light source at a detected central wavelength of ~800 nm. Light emitted by a single mode photonic crystal fiber passes through a collimator and a cylindrical lens to generate a line of light focused on the skin and on a plane reference surface by the microscope objectives (UMPLFLN 20XW, water immersion, 20X, numerical aperture of 0.5, Olympus, Tokyo, Japan). The image of this line is projected on the sensor of a line-scan camera (Octoplus, 2048 pixels, Teledyne e2v, Chelmsford, UK). Silicone oil is used as an immersion medium with a refractive index of 1.4, close to the mean refractive index of skin. A 500 μm thick glass window is placed under each microscope objective to stabilize the skin in the sample arm and provide a low-reflectivity (3.5%) plane reference reflector in the reference arm. When imaging the skin, a film of paraffin oil is deposited between the skin and the glass window to provide refractive index matching between the window and the skin, diminishing the specular back-reflection from both the skin surface and the glass window. The whole interferometer is mounted on a piezoelectric (PZT) linear translation stage (P625.1CD, Physik Instrument, Karlsruhe, Germany) for scanning into the depth (z) of the skin. The interferometer sample arm includes a mirror galvanometer (6210H, Cambridge Technology, Bedford, MA, USA) for lateral (y) scanning of the line of light for en face imaging (horizontal imaging mode). The reference reflector is attached to a piezoelectric (PZT) chip, which can oscillate to generate a phase modulation for en face imaging.

An auxiliary imaging system is incorporated in the LC-OCT device for imaging the skin surface like a dermoscope (see Figure 2). A ring of light-emitting diodes (LEDs) surrounds the microscope objective in the sample arm to illuminate the skin with white light. A beamsplitter mounted on the galvanometer scanner separates the light used for LC-OCT and the light used for dermoscopy. The light transmitted by the dichroic filter is collected by an afocal optical system and sent towards a color-area camera equipped with a micro-objective. The focus of the micro-objective is adjusted dynamically and automatically, so that the image of the skin surface is always in focus, even when the microscope objective is scanned in depth for the LC-OCT image acquisitions. The LC-OCT images and the dermoscopy images are acquired simultaneously and are intrinsically co-localized as they are acquired using the same microscope objective. The field of view of the surface image is 2.6 mm diameter disk. The resolution of the surface image is 5 µm, which enables the observation of the same features as in conventional dermoscopy and therefore provides a direct link between the LC-OCT examination and the dermoscopic examination. The LC-OCT images can thus be acquired in regions of interest identified with the integrated dermoscopy modality.

### 2.2. The LC-OCT Imaging Modes

#### 2.2.1. Vertical Imaging

A vertical section LC-OCT image is acquired by activating the PZT stage to scan into the depth of skin tissues. The PZT stage oscillates at a frequency of 8 Hz according to asymetrical sawthooths with a duty cycle of 80%. The effective amplitude of the scanned depth is Z=400 μm. The camera frame rate is set to 70 kHz so that the step between two consecutive lines acquired by the camera during the displacement of the PZT stage is δ=70 nm, corresponding to a phase-shift of π/2. A stack of Z/δ=5700 lines is acquired during each positive slope of the depth scan. A vertical section image is generated by applying a five-frame fringe envelope detection algorithm [26] to the acquired stack of lines. Acquisition and data processing is repeated continuously during the oscillation cycles of the PZT stage. The resulting images are displayed at a rate of 8 frames/s with automatic contrast optimization following appropriate rescaling. The size of each vertical section image is 2048 × 680 pixels (x×z), corresponding to a field of view of 1.2 mm × 0.4 mm (x×z). The axial resolution (z direction) and the lateral resolution (x direction) are identical, equal to 1.3 µm. The angle of the mirror galvanometer can be controlled by the scroll wheel on the probe handle to change the lateral (y) position of the vertical section. This allows for navigation laterally through the sample being imaged, in real time, with a positioning accuracy far superior to that obtained by moving the probe manually.

**Figure 3:**
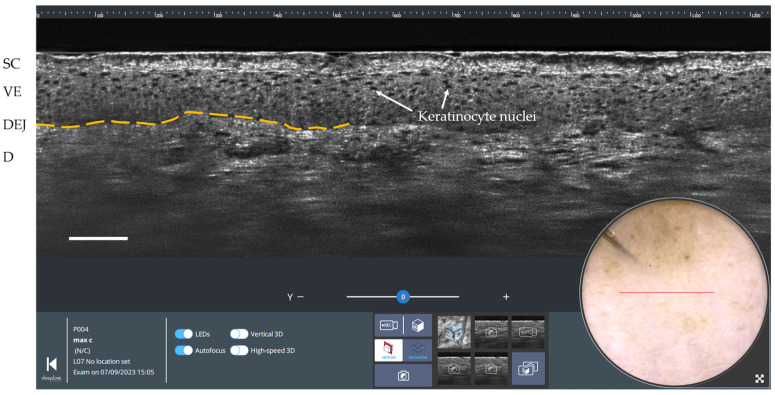
Vertical LC-OCT and dermoscopy image of healthy skin using deepLive™ software. Scale bar: 100 µm. The red horizontal line superimposed on the dermoscopic image delineates the location of the vertical sectional LC-OCT image. SC: stratum corneum; VE: viable epidermis; DEJ: dermal-epidermal junction (partially marked by the yellow dashed line); D: dermis.

An example of a vertical cross-sectional LC-OCT image of healthy skin (forearm of a 25-year-old woman) is shown in Figure 3. The vertical section view enables the visualization of the upper layers and structures of the skin at a cellular resolution, similar to conventional histology. The stratum corneum (SC), viable epidermis (VE), and dermis (D) can be differentiated with the clear dermal-epidermal junction (DEJ). Keratinocyte nuclei are revealed in the viable epidermis. On the surface image, a red line indicates where the LC-OCT vertical section is being acquired.

#### 2.2.2. Horizontal Imaging

In the horizontal imaging mode, the mirror galvanometer operates by executing asymmetrical sawtooth oscillations at a frequency of 8 Hz. These oscillations serve to laterally scan the illumination line (in the y-axis direction) across a 500 µm field. The camera frame rate is set to 100 kHz. Two consecutive lines acquired by the camera are thus separated by a lateral distance of 50 nm. A stack of 10,000 lines is acquired during each positive slope of the lateral scan. The reference reflector of the interferometer is attached to a PZT chip, which oscillates sinusoidally to generate a phase modulation. The frequency and amplitude of the PZT chip oscillation are empirically set at 8 kHz and 1 µm, respectively. A phase-shifting algorithm with sinusoidal phase modulation is used to extract the envelope of the interference fringes [27]. This algorithm processes an algebraic combination of four consecutive lines acquired by the camera to obtain each line of the horizontal section image. The acquisition and data processing procedures are continuously repeated as the mirror galvanometer oscillates back and forth. The horizontal section images are displayed in real time at 8 frames/s with automatically optimized contrast after being appropriately rescaled. The size of each horizontal section image is 2048 × 850 pixels (x×y), corresponding to a field of view of 1.2 mm × 0.5 mm (x×y). The lateral resolutions in the x and y directions are similar, around 1.3 µm. For user convenience, the imaging depth can be adjusted from 0 to 500 µm using the scroll wheel on the probe handle.

**Figure 4:**
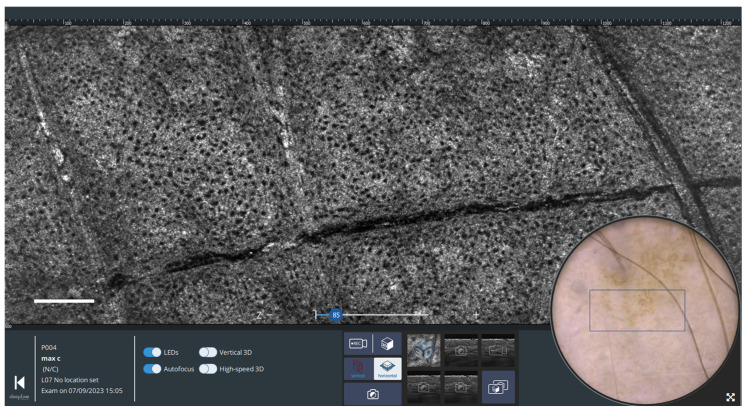
Horizontal LC-OCT and dermoscopy image of healthy skin using deepLive™ software. Scale bar: 100 µm. The blue rectangle superimposed on the dermoscopic image delineates the location of the horizontal sectional LC-OCT image.

A horizontal section LC-OCT image of healthy skin (forearm of a 25-year-old woman) is shown in Figure 4. This image, acquired at a depth of 35 µm, corresponds to a section through the epidermis (stratum spinosum). The keratinocyte nuclei and the honeycomb architecture of the keratinocytes can be clearly visualized. On the surface image, a blue rectangle indicates where the horizontal LC-OCT image is acquired.

#### 2.2.3. Three-Dimensional Imaging

Three-dimensional (3D) images can be generated in less that 25 s from a stack of horizontal sections acquired at successive depths with a step of 1 µm. The resulting 3D image of 2048 × 850 × 500 pixels (x×y×z) is rescaled to 1200 × 500 × 500 pixels for a proper aspect ratio, corresponding to a volume of 1.2 × 0.5 × 0.5 mm^3^ (x×y×z). In practical usage, the 3D imaging mode is selected by the user through the probe handle. By clicking the scroll wheel and turning it in the appropriate direction, the user can choose between 3D image acquisition and video recording. Once 3D acquisition has started, both the PZT stage and the mirror galvanometer move continuously until the PZT stage reaches a depth of 500 µm. When 3D acquisition is complete, the system reverts to the vertical or horizontal imaging mode prior to 3D acquisition.

**Figure 5:**
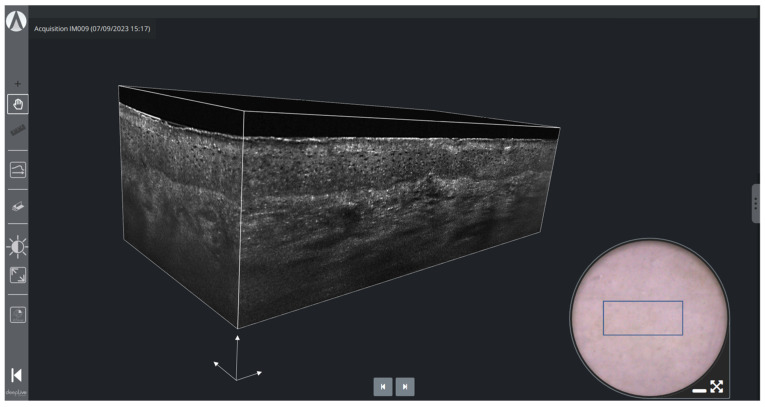
3D LC-OCT rendering and dermoscopy of healthy skin using deepLive™ software. The blue rectangle superimposed on the dermoscopic image delineates the location of the stack of horizontal sectional LC-OCT images. White scale arrows: 100 µm.

Figure 5 shows a 3D LC-OCT image of healthy skin (forearm of a 25-year-old woman). The volume-rendering visualization makes it possible to observe the three-dimensional architecture and connections of the structures in the skin at cellular level, which is not accessible by conventional histology. On the surface image, a blue rectangle indicates where the 3D image is acquired, allowing for easy positioning by the user.

## 3. Results and Discussion

The imaging protocol begins with the application of a drop of paraffin oil on the skin. Next, the LC-OCT probe is gently pressed against the skin, flattening and stabilizing the area to be imaged. The dermoscopy image serves first to target the lesion, then to guide the examination and ensure sufficient coverage of the lesion. Although the extent of skin lesions generally exceeds the field of view of LC-OCT, real-time image acquisition in vertical and horizontal mode at 8 frames per second combined with the guiding surface image allows for the quick and easy coverage of the entire lesion. The appropriate imaging mode depends on the expected nature of the lesion. The vertical mode is well-suited for assessing the presence of lobular structures and their position relative to the epidermis (connected or separated), the appearance of the dermo-epidermal junction (preserved or destroyed), and the position of cellular atypia in the epidermis (close to the basal layer or in pagetoid ascent). The horizontal mode is more effective for analyzing the regularity of the keratinocyte network or revealing the presence of dendritic cells, which are preferentially distributed in a horizontal plane parallel to the skin surface. Consequently, the vertical mode is mainly used to diagnose basal cell carcinomas (BCCs), actinic keratoses (AKs), squamous cell carcinomas (SCCs), or inflammatory lesions, while the 3D mode is preferred for atypical melanocytic lesions, as both vertical and horizontal analyses provide complementary information.

### 3.1. Melanocytic Skin Tumors

**Figure 6:**
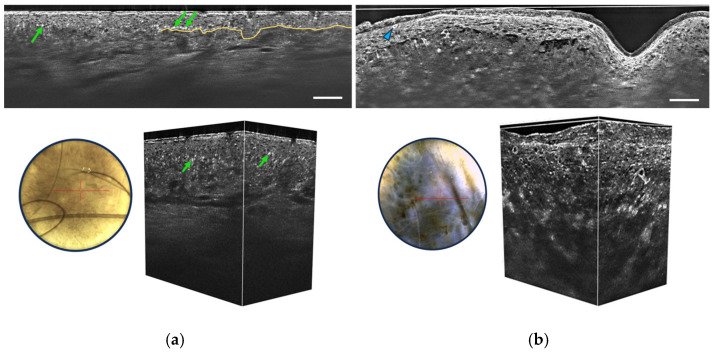
Vertical sectional LC-OCT image, dermoscopic image (left), and 3D LC-OCT reconstruction (right) of a junctional nevus (**a**) and a melanoma (**b**). The red horizontal line superimposed on the dermoscopic images delineates the location of the sectional LC-OCT images. (**a**) Regular organization of keratinocytes in the viable epidermis, pigmentation appearing as white discs (green arrows), well-defined DEJ partially marked by the yellow line. (**b**) Disorganized epidermis, pagetoid cells (blue arrow), atypical cells in the dermis, and damaged DEJ. Scale bars: 100 µm.

Regarding the diagnosis of melanocytic lesions, LC-OCT detection criteria associated with melanoma include the following [28,29]:

- Irregular honeycombed pattern: Melanomas often exhibit irregular honeycombed structures in LC-OCT, characterized by disorganized, unevenly spaced, and variably sized cells nuclei, characterized by a network of roundish hyporeflective structures on LC-OCT images.
- Pagetoid spread: LC-OCT allows for the visualization of pagetoid spread, which refers to the presence of melanocytes invading the upper layers of the epidermis in a solitary or nested pattern (Figure 6b). This epidermal invasion is a hallmark feature of melanomas and is not typically observed in benign nevi.

The absence of these criteria throughout the lesion is strongly associated with benign melanocytic lesions, in which melanocytes may form clusters at the junction and/or in the dermis. Some benign dermal melanocytic proliferations may also show a “wave-like pattern” on LC-OCT, characterized by alternating undulated hyper-reflective and hypo-reflective lines in the papillary and reticular dermis, corresponding to the compression of collagen fibers around low-pigmented melanocytic nests [30]. Other criteria for benign melanocytic proliferations include a well-outlined dermal-epidermal junction (DEJ) and the absence of atypical cells in the epidermis and dermis (Figure 6a).

The performance of LC-OCT in assessing melanoma versus nevi has been shown to be similar to that of RCM [28]. A high correlation with histopathology and RCM was found in the characterization of different types of melanocytic lesions, such as lentigo maligna [29,31].

### 3.2. Non-Melanocytic Skin Tumors

#### 3.2.1. Basal Cell Carcinoma

**Figure 7:**
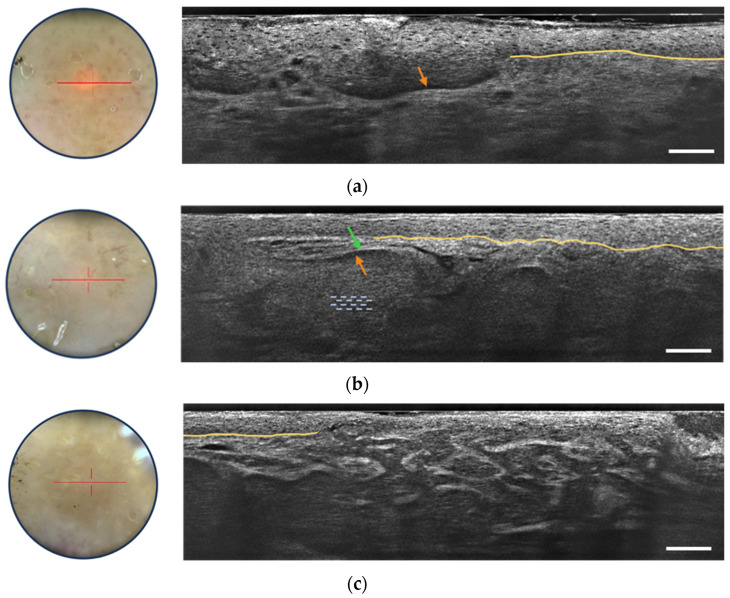
Dermoscopic image (left) and vertical sectional LC-OCT image (right) of a superficial basal cell carcinoma (BCC) (**a**), a nodular BCC (**b**) and an infiltrative BCC (**c**). The core of the lobule is characterized by a millefeuille pattern (blue dotted lines) delimited by its clefting (orange arrow) and bright rim (green arrow). The yellow line represents the partially drawn DEJ. The red horizontal line superimposed on the dermoscopic image delineates the location of the vertical sectional LC-OCT image. Scale bars: 100 µm.

LC-OCT provides valuable criteria for the detection of basal cell carcinoma, particularly focusing on the visualization of dermal lobules (see Figure 7) [32]. Among these criteria, three distinct components within a lobule are identified as the most important for BCC recognition:

- Grey core with millefeuille pattern: This component appears as a laminated structure oriented along the horizontal plane, resembling the layers of the French pastry “millefeuille”. It represents the dense cellularity within the basaloid tumor island and includes basaloid and immune cells.
- Middle dark rim (clefting): Immediately surrounding the grey core of the lobule, the middle dark rim is referred to as “clefting” and likely corresponds to the peritumoral mucin deposition [33].
- Outer bright rim: This rim results from the compression and alteration of collagen fibers in the stroma due to the presence and interaction of the tumor island, creating a mass effect.

This detailed characterization of the core of the BCC lobule is possible due to the high-resolution, high-penetration, and vertical/3D visualization capabilities of LC-OCT. The simultaneous presence of the inner lamination, middle clefting, and outer bright rim aids in the differentiation of BCCs from other cutaneous lesions displaying dermal lobules. Moreover, LC-OCT allows for the subtyping of BCCs based on lobule shape and position [34]. For instance, hemispheric lobules connected to the epidermis are associated with superficial BCC (Figure 7a), while macrolobules separated from the epidermis are characteristic of nodular BCC (Figure 7b), and branched lobules in the dermis are indicative of infiltrative BCC (Figure 7c). These criteria are useful in real-life clinical situations for correct BCC diagnosis and subtyping without the need for biopsy, enabling appropriate therapeutic decisions to be taken immediately. The integration of LC-OCT in dermatological practice empowers practitioners with improved diagnostic confidence and efficiency in managing BCC cases. The LC-OCT ability to provide additional information, complementing dermoscopy, makes it a valuable non-invasive tool for accurate BCC detection and subtyping [35]. A recent three-year study has shown the potentially important role of LC-OCT in the non-invasive diagnosis of non-melanocytic skin tumors, particularly BCC, avoiding unnecessary biopsies with increased sensitivity, much higher specificity, and better accuracy than clinical assessment with dermoscopy alone [36].

#### 3.2.2. Actinic Keratosis and Squamous Cell Carcinoma

**Figure 8:**
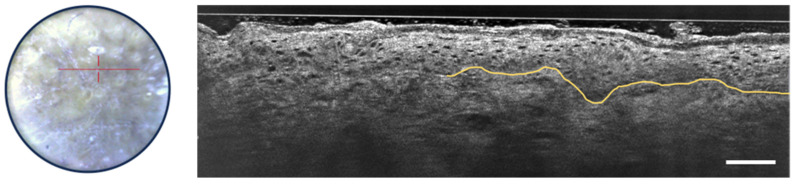
Dermoscopic image (**left**) and vertical sectional LC-OCT image (**right**) of an actinic keratosis (AK). Unusual organization, size and shape of keratinocyte nuclei within the epidermis, hyperkeratosis, undulated but still defined DEJ, partially drawn in yellow. The red horizontal line superimposed on the dermoscopic image delineates the location of the vertical sectional LC-OCT image. Scale bar: 100 µm.

LC-OCT has shown promise in diagnosing and distinguishing squamous cell carcinoma and actinic keratosis. Several studies [37,38] have identified LC-OCT criteria for AK, including hyperkeratosis (irregular, hyper-reflective stratum corneum), cellular and nuclear pleomorphism affecting basal and upper epidermal layers and a well-defined DEJ along the entire lesion as can be seen in Figure 8. The ability of LC-OCT to visualize the basal growth pattern of AK enables non-invasive classification based on the histological PRO classification. Comparing AK and SCC, both exhibit cytological and architectural alterations, such as epidermal pleomorphism, cellular atypia, and dilated and glomerular vessels [39,40]. Nevertheless, AK is associated with a preserved DEJ, whereas SCC shows a non-visible or interrupted DEJ [40]. LC-OCT outperformed RCM in visualizing key features of keratinocyte skin tumors, including parakeratosis, dyskeratotic keratinocytes, and dilated and glomerular vessels [41]. Overall, LC-OCT holds significant potential for the real-time, non-invasive diagnosis and subtyping of AK and SCC and its advantages over RCM highlight its clinical utility.

**Figure 9:**
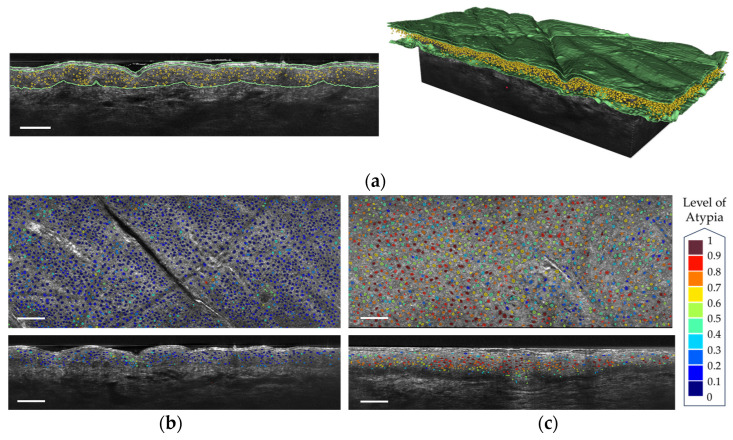
(**a**) Segmentation of the keratinocyte nuclei and of the epidermis layers in a vertical LC-OCT image (**left**) and a 3D reconstruction (**right**). (**b**) Segmentation of the keratinocyte nuclei colored according to their level of atypia on healthy skin (**c**) Segmentation of the keratinocyte nuclei colored according to their level of atypia on an AK. Scale bar: 100 µm.

DAMAE Medical has developed deep learning algorithms for the segmentation of skin layers and keratinocyte nuclei in LC-OCT images. These algorithms enable the automatic segmentation of the stratum corneum, the viable epidermis, and the dermis. The segmentation of the viable epidermis and dermis yields a segmentation of the dermal-epidermal junction (DEJ). Artificial intelligence using convolutional neural networks was demonstrated to assess the malignant potential of AK by grading the undulation of the DEJ [42]. Automated PRO score models were developed in accordance with the histopathological gold standard [43,44,45] for assessment in the follow-up of AK in vivo from LC-OCT images. The segmentation of keratinocyte nuclei in a 3D LC-OCT image can be performed in less than two minutes, whereas it is infeasible by hand since a single 3D LC-OCT image can count more than 40,000 cells. Figure 9a shows an example of segmentation of both the skin layers and the keratinocyte nuclei. The output segmentations of keratinocyte nuclei can enable the assessment of atypia at the cellular level, which is a complex challenge that could aid in the early diagnosis and monitoring of keratinocyte cancers [46]. The segmentations are utilized to calculate cellular-level metrics including nucleus volume, compactness, and number of neighboring cells. Subsequently, a semi-supervised machine learning model [47], based on these metrics, has been developed to predict cellular atypia both at the cellular level and in 3D image analysis. This approach allows for the differentiation of pathological skin from healthy skin, as illustrated in Figure 9b,c for AK. This algorithm was demonstrated to have the potential to grade pre-cancerous keratinocytic lesions and help in treatment monitoring [48].

#### 3.2.3. Inflammatory and Infectious Skin Diseases

**Figure 10:**
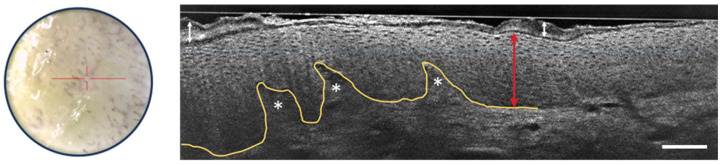
Dermoscopic image (**left**) and vertical sectional LC-OCT image (**right**) of psoriasis. Thickening of the stratum corneum (white arrow with two heads) and of the viable epidermis (red arrow with two heads) with elongated dermal papillae (white asterisks). Keratinocytes nuclei are well organized. Undulated but still well-defined DEJ, which is partially drawn in yellow. The red horizontal line superimposed on the dermoscopic image delineates the location of the vertical sectional LC-OCT image. Scale bar: 100 µm.

Recent studies and case reports have shown that LC-OCT is a valuable tool for diagnosing various inflammatory and infectious skin conditions, focusing on architectural criteria such as fluid accumulation, changes in epidermal layers, the shape of the DEJ, vascular architecture, the presence of inflammatory infiltrates, and the detection of foreign bodies [49,50,51,52,53]. Figure 10 shows some of the LC-OCT detection criteria of psoriasis. The stratum corneum shows hyperkeratosis. The viable epidermis is also thicker than usual, but the nuclei of keratinocytes are organized regularly. The DEJ is well-defined and undulates due to elongated dermal papillae.

Further advancements in image analysis software and artificial intelligence could enhance capabilities in quantifying skin metrics, such as layer thickness and DEJ undulation, leading to an improvement in diagnostic accuracy and treatment monitoring of inflammatory skin diseases [54].

## 4. Conclusions

LC-OCT is becoming a technique of choice for non-invasive skin imaging at high resolution, especially for the early diagnosis and therapeutic follow-up of many skin diseases including cancers. In the form of a handheld probe, the deepLive™ device developed by DAMAE Medical is of convenient use by dermatologists, providing easy access to almost any part of the skin. LC-OCT offers imaging capabilities with a cellular resolution (~1 µm) closely approaching that of RCM, with the advantage of being able to generate vertical and horizontal sections as well as 3D images, combined with a dermoscopy-like surface image acquired in parallel. LC-OCT images have an accessible field of view of ~1.2 mm × 0.5 mm × 0.5 mm (x×y×z), smaller than the field of view of conventional OCT images but with the advantage of better resolution. The vertical and horizontal sections are displayed in real-time at 8 frames per second, and the 3D images are acquired in less than 25 s. The surface images, with a resolution of ~5 µm, allows practitioners to easily locate where to acquire LC-OCT images based on dermoscopic examination. An efficient workflow has been developed and implemented in the software associated with deepLive™ for easy usage in clinical routine, help with margin assessment and easy patient management for follow-up examinations [25].

As LC-OCT can generate a large amount of data, automated deep learning algorithms have been developed to save time when examining and annotating images, and even to perform tasks that cannot be carried out manually within a reasonable timeframe. Using LC-OCT images, artificial intelligence is able to quantify keratinocyte atypia and DEJ undulation in AK and SCC, providing standardized severity assessment and treatment monitoring in the cancerization field. DAMAE Medical is currently developing deep learning algorithms capable of detecting and diagnosing lesions in real time during image acquisition. These artificial intelligence algorithms will provide guidance during live examinations to take images more efficiently and quickly, helping novice users. Ultimately, artificial intelligence algorithms should significantly improve diagnostic accuracy. Artificial intelligence will also facilitate the creation of new indications for patient follow-up, such as margins assessment before surgery (whether for lentigo maligna, SCC, or BCC) and the follow-up of non-invasive treatments for BCC, AK, and inflammatory pathologies.

By measuring the intensity of light backscattered by tissue micro-structures, LC-OCT is able to reveal the internal morphology of the skin down to the cellular level. This mode of contrast, resulting from variations in the tissue refractive index, cannot match the specificity of histology, where histochemical stains with hematoxylin and eosin, or immunohistochemical stains such as HER2 or Ki-67, enable tissue structures and cellular details to be unequivocally differentiated. However, histology is destructive and costly in terms of time and resources. Moreover, histology can only assess tissue distribution in 2D, with a loss of information due to the restricted orientation of the sections. In addition, samples have to be embedded before sectioning, which can lead to tissue deformation and thus distort structural tissue analysis. In contrast, LC-OCT enables the 3D visualization of tissue structures non-invasively, avoiding any risk of deformation. We believe that the relative lack of specificity of LC-OCT compared with histology could be compensated for, at least in part, by AI-based image analysis. On the other hand, the coupling of LC-OCT with spectroscopic techniques, such as Raman scattering spectroscopy, is another approach currently under development [55], enabling information on the molecular composition of tissues to be obtained in addition to purely morphological information, thus increasing the specificity of examinations.

## Data Availability

Fully anonymized data are available upon request.
